# Dynamics of crevice microbubbles that cause the twinkling artifact

**DOI:** 10.1016/j.ultsonch.2024.106971

**Published:** 2024-06-25

**Authors:** Eric Rokni, Eusila C. Kitur, Julianna C. Simon

**Affiliations:** Graduate Program in Acoustics, The Pennsylvania State University, University Park, PA 16802, USA

**Keywords:** Microbubbles, Twinkling artifact, Silicon, Cavitation

## Abstract

The Doppler ultrasound twinkling artifact, a rapid color shift, appears on pathological mineralizations and is theorized to arise from scattering off micron-sized crevice microbubbles. However, the influence of crevice number and size as well as the bubble dynamics on twinkling is not well-understood. Cylinders with diameters of 0.8–1.2 µm and depths of 1 µm were etched into a silicon wafer and crevice bubbles were driven at 0.75, 2.5, and 5.0 MHz while monitoring with high-speed photography. Experimental results were compared to a derived crevice bubble model. On three separate wafers, cylindrical crevices (10 or 100) with diameters of 1, 10, or 100 µm and depths of 10 µm were etched and imaged with a research ultrasound system in Doppler mode at 5, 7.8, and 18.5 MHz. Within the pressure ranges studied here (∼1 MPa), no bubble oscillation was observed for the 0.8–1.2 µm crevice bubbles which matched computational results. Crevices with 1 and 10 µm diameters produced more twinkling than 100 µm crevices at 5 and 7.8 MHz. In contrast, 100 µm crevices produced more twinkling than 1 or 10 µm crevices at 18.5 MHz (p < 0.001 in all cases). These results provide better insight into how crevice bubbles cause twinkling on pathological mineralizations.

## Highlights

• Modifying the Rayleigh-Plesset equation to include cylindrical boundaries gives insight into the behavior of crevice bubbles that cause twinkling.
• Micron sized crevice bubbles on silicon do not appear to grow in the presence of an ultrasound field.
• Twinkling is affected by the number and size of crevice bubbles, with even 10, 1 µm diameter crevices etched on silicon producing twinkling.

## 1 Introduction

Acoustic cavitation in a bulk liquid typically arises from heterogenous nuclei (i.e. from preexisting gas pockets within the medium). These heterogeneous nuclei have been modeled as microbubbles in crevices on motes, stabilized by organic impurities, or near hydrophobic surfaces [1], [2]. In 2013, the crevice bubble theory of nucleation was used to explain the mechanism of the color Doppler ultrasound twinkling artifact, or twinkling [3]. Twinkling appears on hard mineralizations as a rapid color shift and has been used to improve the detection and diagnosis of pathological mineralizations [4], [5], [6], [7], [8], [9], [10], [11], [12], [13], [14]. Previous studies have provided strong experimental and visual evidence that bubbles are present on mineralizations and cause twinkling [3], [15], [16]. However, the mechanisms by which bubbles cause twinkling are not fully understood. Here, we experimentally evaluate the effect of crevice size and number on twinkling in etched silicon wafers and compare observed bubble dynamics to a computational model.

Although bubbles are believed to cause twinkling, the location, size, and number of bubbles on pathological mineralizations remain unknown. Macroscopic surface roughness has been linked to twinkling as rougher kidney stones tend to produce more twinkling [17], [18], [19], [20], [21]. However, other studies found no correlation between increased macroscopic surface roughness and twinkling, with even smooth materials twinkling [15], [16]. These discrepancies led researchers to evaluate microstructures on kidney stones and other mineralizations, which showed complicated arrays of 1–100 µm surface crevices [16], [22]. Environmental scanning electron microscopy on kidney stones revealed water first condensed on the smallest crevices (1 µm) as humidity increased, suggesting that the smallest crevices preferentially stabilized bubbles [16]. However, the exact influence of different sized and shaped crevice bubbles on twinkling remains unclear, necessitating the development of accurate computational models.

While models have been developed to evaluate how crevices stabilize bubbles against dissolution [1], [2], the dynamics of crevice bubbles in an ultrasound field has not been fully modeled. The classic Rayleigh-Plesset (RP) equation was developed in 1917 from first principles to describe how the radius of a single, spherically symmetric bubble responds to an external pressure function over time [23], [24], [25]. Many modifications of the RP equation have been developed that include free non-spherical bubbles, bubbles in highly viscous media, and bubbles at a wall boundary [26], [27], [28]. There are several ways to approach the problem of added boundaries. On first order, Bremond et al. (2006) [28] used an equivalent radius in the traditional RP equation and found the model generally tracked well with experimental results of a collapsing bubble at a boundary. Computational Flow Dynamic (CFD) models have also been developed to include boundaries for collapsing bubbles (ECOGEN) [29], [30] and corresponded to the growth and collapse of artificial bubbles on kidney stones; however, these models have only been explored for a singular collapse and can be computationally expensive. Leighton et al. (2000) [31] derived an equation to include the boundaries of a conical bubble through energy balance, but the model is only applicable for the collapse of a conical bubble through a custom U-tube apparatus. To accurately predict the dynamics of crevice bubbles responsible for twinkling, further modifications to these existing models are necessary. These modifications could also be useful in understanding the behavior of spherical bubbles constrained inside cylindrical blood vessels [32].

In this work, a RP-like equation was derived to include cylindrical crevice boundaries. The model was compared to experimental results from crevices with diameters of 0.8–1.2 µm driven with ultrasound and imaged through an inverted microscope with high-speed photography. Then, 10 or 100 randomly-spaced cylindrical crevices with diameters of 1, 10, or 100 µm were etched on silicon wafers and imaged with Doppler ultrasound to investigate the effect of crevice number and size on twinkling.

## 2 Theory

The classical RP equation[23], [24], [25] for a spherical bubble can be derived through energy balance to be

(1) $$R\ddot{R}+\frac{3}{2}{\dot{R}}^{2}=\frac{1}{\rho_{0}}\left[\left(p_{0}+\frac{2\sigma }{R_{0}}-p_{v}\right){\left(\frac{R_{0}}{R}\right)}^{3\kappa }+p_{v}-\frac{2\sigma }{R_{0}}-p_{0}-P\left(t\right)\right]$$

where *R* is the time varying bubble radius, $\rho_{0}$ is the density of the liquid, *p_0_* is the ambient pressure, σ is the surface tension, *R_0_* is the initial bubble radius, κ is the polytropic index, $p_{v}$ is the vapor pressure, and P(t) is a time varying external pressure function. Introducing cylindrical crevice boundaries modifies the standard RP equation by changing the balanced energies. The kinetic energy (KE) is related to the density, volume, and velocity of the bubble wall [25], and can be given in cylindrical coordinates as

(2) $$KE_{\mathrm{cyl}}=\pi a\rho_{0}\int_{R}^{\infty }\frac{{\dot{R}}^{2}R^{2}}{r}dr,$$

where *a* is the width of the cylinder. This integral does not converge when evaluated infinitely far away which limits the the boundaries to a finite distance[33], *r_0_*, related to the driving frequency and initial bubble radius. This integral simplifies to,

(3) $$KE_{\mathrm{cyl}}=\pi a\rho_{0}{\dot{R}}^{2}R^{2}ln\left(\frac{r_{0}}{R}\right)$$

The work done by the bubble wall is related to the pressure far from the bubble ($p_{\infty }$) and the pressure exerted on the bubble wall from the surrounding liquid ($p_{L}$) and is

(4) $$W={(p}_{\infty }-p_{L})\int_{R_{0}}^{R}2\pi aRdR.$$

Equating Eqs. (3), (4) and differentiating with respect to *R* gives

(5) $$\left({\dot{R}}^{2}+R\ddot{R}\right)ln\left(\frac{r_{0}}{R}\right)-\frac{1}{2}{\dot{R}}^{2}=\frac{{(p}_{\infty }-p_{L})}{\rho_{0}}.$$

The liquid pressure immediately outside the bubble wall can be written as [34]

(6) $$p_{L}=\left(\left(p_{0}+\frac{\sigma }{R_{0}}+\frac{2\sigma }{a}-p_{v}\right){\left(\frac{R_{0}}{R}\right)}^{2\kappa }+p_{v}-\frac{\sigma }{R_{0}}-\frac{2\sigma }{a}\right),$$

while the pressure far from the bubble is

(7) $$p_{\infty }=p_{0}+P\left(t\right).$$

This results in the modified RP equation for bubbles in cylindrical crevices,

(8) $$\left({\dot{R}}^{2}+R\ddot{R}\right)ln\left(\frac{r_{0}}{R}\right)-\frac{1}{2}{\dot{R}}^{2}=\frac{{(P\left(t\right)+p}_{0}-\left(p_{0}+\frac{\sigma }{R_{0}}+\frac{2\sigma }{a}-p_{v}\right){\left(\frac{R_{0}}{R}\right)}^{2\kappa }-p_{v}+\frac{\sigma }{R_{0}}+\frac{2\sigma }{a})}{\rho_{0}}.$$

The above derivation makes many assumptions: the crevice bubble is in an infinite medium, gravity and bulk viscosity can be ignored, there is no mass loss from the bubble, vapor pressure is constant, the bubble is now cylindrical, and it does not consider the bottom of the crevice.

## 3 Materials and methods

### 3.1 Silicon fabrication

Silicon wafers with 2″-diameter, 279 ± 25 µm thick, with 〈1 0 0〉 orientation and 1–10 O-cm resistivity were used for all experiments (Nova Electronic Materials, Flower Mound, TX, USA). To fabricate crevices on the wafers, the wafer was firstspin coated with hexamethyldisilazane (HMDS) and baked at 105 °C for 60 sec followed by SPR 955 photoresist (Kayaku Advanced Materials, Westborough, MA, USA) at 4500 rpm for 90 s and baked at 105 °C for 60 sec. Designs made in KLayout (GDS2 Viewer, Munich, Germany) were then exposed onto the photoresist using an MLA 150 Direct Write Exposure Tool (Heidelberg, Germany). After exposure, the wafer was developed in CD-26 (TMAH) for 60 s, rinsed with deionized (DI) water, and dried with N_2_. Etching was performed using the Haber-Bosch process (SPTS LPX Deep Silicon Etch, Ringland Way, Newport, UK). The photoresist was then stripped off using PRS-3000 at 80 °C and the samples were cleaned with isopropyl alcohol and DI water. This process was performed in a nanofabrication cleanroom at the Pennsylvania State University Materials Research Institute.

**Fig. 1:**
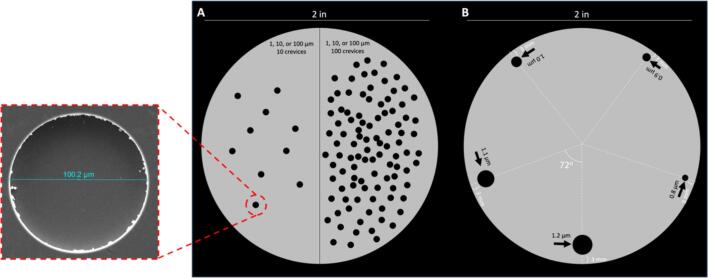
Silicon lithography mask designs for (A) imaging crevice bubbles on silicon and (B) evaluating twinkling on silicon. Features in white are guide-lines while features in black are the designs that were etched. Drawing is not to scale to allow for visibility of features. The inlay with red dashed lines is a representative scanning electron microscopy image of a 100 µm diameter crevice. (For interpretation of the references to color in this figure legend, the reader is referred to the web version of this article.)

On one wafer, five cylindrical crevices with diameters of 0.8–1.2 μm and depths of 1 μm (Fig. 1a) were dry etched 3 mm from the edge of the silicon at 72° increments. On three additional wafers, half the wafer was dry etched with 10 crevices while the other half was etched with 100 crevices; crevices were randomly distributed on each half (Fig. 1b). The random distribution was chosen to avoid any unintended interference effects of a symmetrical layout and to more accurately mimic how crevices might appear on a mineralization. The crevices were either 1, 10, or 100 µm diameter and 10 µm deep. To approximate the surface tension between the silicon, water, and air, contact angle measurements [35] were made using a Canon Rebel T6 camera (Canon U.S.A, Inc., Melville, NY) resulting in a surface tension of ∼3000 mN/m.

### 3.2 Imaging crevice bubbles on silicon

**Fig. 2:**
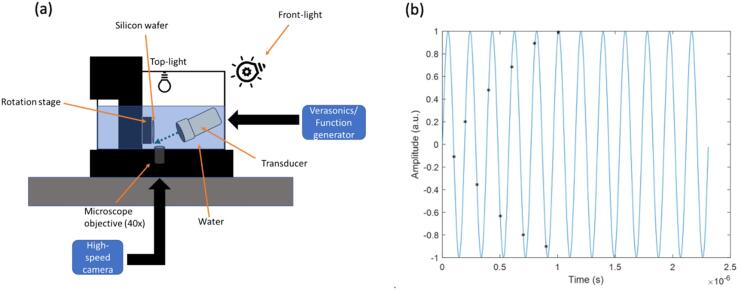
(color online) (a) Diagram of the experimental arrangement for high-speed imaging of crevice bubbles on silicon. (b) Example of frame captures (*) when driving at 5.0 MHz and capturing frames at 200 fps. Delays of 5–6 µs were applied to allow for interleaving of camera frames.

The wafer design shown in Fig. 1a was used to image the dynamics of crevice bubbles formed on silicon wafers. Wafers were submerged in a small tank filled with degassed, DI water (<2 mg/L oxygen content; Extech D0210 Dissolved Oxygen Meter, Extech, Waltham, MA, USA). Crevice bubbles were located using an inverted microscope (Nikon Ti2-U, Melville, NY, USA) with an N40X-NIR objective (3.5 mm working distance; Nikon, Tokyo, Japan) (Fig. 2a). A rotation stage (Newport RS40, Irvine, CA, USA) was used to adjust the position of the wafer. An L7-4 transducer operating at 5.0 MHz with peak Doppler pressures of *p_+_* ≈ 0.8 MPa and *p_-_* ≈ 0.7 MPa (Philips/ATL, Bothell, WA, USA) or P4-2 transducer at 2.5 MHz with peak Doppler pressures of *p_+_* ≈ 1.0 MPa and *p_-_* ≈ 0.9 MPa (Philips/ATL, Bothell, WA, USA) were aligned with the wafer and transmitted a Doppler pulsing scheme in plane wave mode consisting of 12 ensembles of 12 cycles repeated every 3000 Hz. Peak pressures were measured in the small tank with a golden capsule hydrophone (HGL-Series, Onda, Sunnyvale, CA, USA). Additionally, a custom-built focused transducer at 750 kHz with f# = 1 and peak pressures of *p_+_* ≈ 3 MPa and *p_-_* ≈ 1.5 MPa (measured in bulk water) was also used. The exposure parameters ranged from 0.2 ms pulses repeated every second up to continuous waves. The pressure of the custom transducer was not measured in the small tank microscope setup, but are assumed to be similar to the imaging transducers. These transducers were chosen in an attempt to produce the largest response from the bubble. Due to the small tank setup, transducers were angled for alignment with the bottom crevice in the wafer. A high-speed camera operating at frame rates of 20–200 kfps (Photron FastCam Nova S-9, San Diego, CA, USA) was synchronized with each transducer. A constant time delay was included to account for the travel time between the transducer and silicon wafer; additional varying time delays were used to capture frames at different time points to create an effective sampling rate of 10 Mfps (Fig. 2b). Assuming bubble motion was consistent between acoustic cycles, each captured frame was then stitched together to observe the full motion of the bubble. Lighting for the high-speed camera consisted of a combination of top microscope lighting and a Photogenic Powerlight (2500DR-UV, Bartlett, IL, USA) used in flash mode for front-lighting.

### 3.3 Evaluating twinkling on silicon

Three wafers with the design in Fig. 1b (crevice diameters of 1, 10, or 100 µm) were imaged with a Verasonics research ultrasound system (Vantage, Verasonics®, Kirkland, WA, USA). The same L7-4 transducer operating at 5.0 MHz with peak Doppler pressures of *p_+_* ≈ 3.6 MPa and *p_-_* ≈ 3.1 MPa with an elevation focus of ∼30 mm, along with an L12-5 transducer (Philips, Bothell, WA, USA) operating at 7.8 MHz with peak Doppler pressures of *p_+_* ≈ 3.2 MPa and *p_-_* ≈ 1.3 MPa with an elevation focus of ∼15 mm, or L22-14v transducer (Verasonics®, Kirkland, WA, USA) operating at 18.5 MHz with peak Doppler pressures of *p_+_* ≈ 2.4 MPa and *p_-_* ≈ 1.7 MPa with an elevation focus of ∼10 mm were aligned with the wafer and transmitted plane-wave Doppler pulsing schemes consisting of 12 ensembles of 12 cycles with 3000 Hz pulse repetition frequency. Peak pressures were measured in a large water tank with the golden capsule hydrophone. These linear array transducers had −6 dB azimuthal angles of ∼1.7°, ∼1.1°, and ∼0.7°, respectively, and were chosen to allow for more direct comparison of the effect of frequency on twinkling. The L7-4 transducer at 5.0 MHz was the lowest tested frequency as it provides a good balance between imaging resolution and twinkling amplitude [16]. All ultrasound scans were performed in the large water tank with degassed DI water. The wafers were placed on a block of neoprene and each transducer was centered directly above the side of interest at its focus. Each half of the wafer (10/100 crevices) was imaged for ∼1 min without moving the transducer while saving in-phase quadrature (IQ) data at approximately 2 frames per second. As silicon is a highly reflective surface, it produces a small amount of noise in the Doppler signal when imaged; therefore, a silicon wafer with no crevices etched was also imaged as a control. The Doppler power in a region of interest around the wafer was summed and averaged over the imaging time. This value was divided by the mean value of the unetched control to calculate a normalized Doppler power. Normality was tested for using a Ryan-Joiner test and a General Linear Model with post-hoc Bonferroni tests was performed to determine the effect of size and number of crevices on twinkling with p < 0.05 indicating significance. Statistical analyses were performed in Minitab (Minitab, State College, PA, USA).

### 3.4 Computation

The cylindrical bubble model and RP equation (i.e. spherical bubble model) were entered into MATLAB (Mathworks, Natick, MA, USA) and the ordinary differential equations were solved using *ode45*. The cylindrical crevice bubble model was compared with experimental results found in Ziljstra et al. [36] where a cylindrical crevice bubble with width of 30 µm and depth of 10 µm was driven at 80 kHz for model validation (Table 1). Briefly, the bubble radii from the photographs in Ziljstra et al. that were captured every 2 µs were approximated and plotted against the computational results. Anytime the bubble fell below the crevice opening and could not be visualized, it was plotted as the crevice depth (i.e. 10 µm). The percent difference between the photographed radius and the model was calculated. As the driving amplitude was not specified in Ziljstra et al., an amplitude of 0.15 MPa was arbitrarily used.

**Table 1:** Input computational parameters used to compare with experimental results.

Input parameters	Ziljstra et al [36]	Bubble dynamics	Doppler imaging
Frequency (MHz)	0.08	0.75, 2.5, 5	5, 7.8, 18.5
Amplitude (MPa)	0.15	1	4
Crevice diameter (µm)	30	0.8, 0.9, 1.0, 1.1, 1.2	1, 10, 100
Depth (µm)	10	1	10

After validating the model from the results of Ziljstra et al., the experimental parameters used in the present study and given in Table 1 were then input into the model and compared with the experimental results. Here, the growth of the bubble radius on the y-axis was normalized to the crevice depth and refers to how the bubble expands into or out of the crevice. To help evaluate spectral content of the bubble oscillations, power spectra of the bubble oscillations simulated by the crevice model were calculated with record lengths ranging from 13,000–40,000 samples, depending on the frequency. All simulations assumed the bubbles were composed of air and formed in water on silicon, so the medium density was 1000 kg/m^3^ and the viscosity was 0.001 Pa s. When plotting the results of the cylindrical bubble model, the growth of the bubble radius on the y-axis refers to how the bubble expands into or out of the crevice.

## 4 Results

### 4.1 Imaging crevice bubbles on silicon

**Fig. 3:**
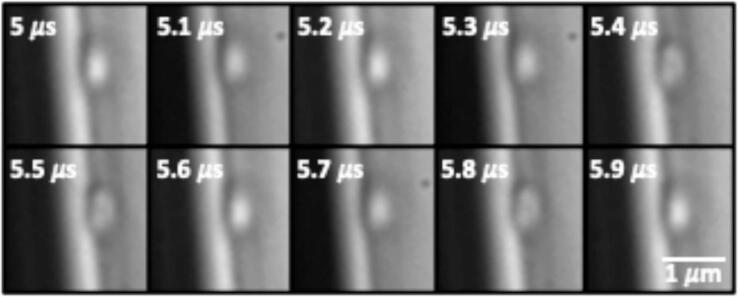
Images of 1.2 µm diameter crevice bubble driven by 5.0 MHz Doppler pulses. Frames were captured at 200 fps with the respective time delays. There was no noticeable change of the bubble radius, which was consistent for all tested parameters.

Bubbles were successfully visualized through the microscope with the high-speed camera for all crevice sizes. For all 5 tested crevice sizes (0.8–1.2 μm), driving with Doppler ultrasound at 2.5 or 5.0 MHz or with the custom transducer at 0.75 MHz caused no visible change in the bubble radius. A representative example is presented in Fig. 3 for the 1.2-μm bubble driven with Doppler ultrasound at 5.0 MHz.

### 4.2 Evaluation of twinkling on silicon

**Fig. 4:**
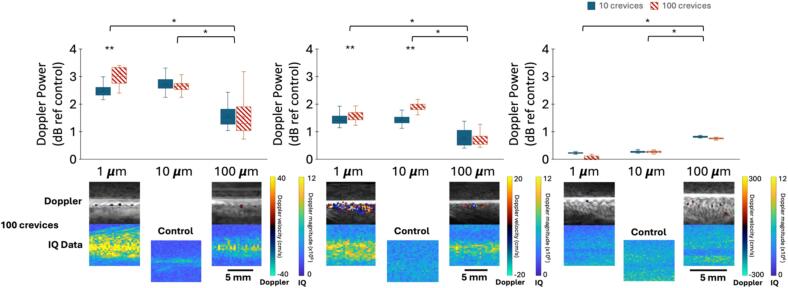
(color online) Boxplots representing the Doppler power on each silicon wafer with reference to the control wafer when imaged at (A) 5.0 MHz (*p_+_* ≈ 3.6 MPa), (B) 7.8 MHz (*p_+_* ≈ 3.2 MPa), and (C) 18.5 MHz (*p_+_* ≈ 2.4 MPa). One asterisk indicates significant differences (p < 0.05) between crevice size while two asterisks represent significant differences (p < 0.05) between number of crevices. Representative frames of the image produced by Verasonics (top) and the image created from the saved IQ data (bottom) for 1 µm and 100 µm (100 crevices) and control are presented for each frequency.

All wafers with any tested number (10 or 100) or size (1, 10, 100 µm) of crevice twinkled (Fig. 4). When imaged at 5.0 MHz, significantly more twinkling (p < 0.001) appeared with 1 or 10 µm crevices compared to 100 µm (Fig. 4a). Additionally, there was a significant increase in twinkling for 1 µm crevices when the number of crevices increased from 10 to 100 (p = 0.005); no difference was observed for number of crevices at 10 µm (p = 0.3) and 100 µm (p = 0.5). Results were similar at 7.8 MHz with significantly higher Doppler power with 1 µm (p < 0.001) or 10 µm (p < 0.001) crevices compared to 100 µm (Fig. 4b). However, at 7.8 MHz, twinkling significantly increased for both 1 µm (p = 0.04) and 10 µm (p < 0.001) crevices when increasing the number of crevices from 10 to 100; there was still no change based on number of crevices at 100 µm (p = 0.1). At 18.5 MHz, twinkling was highest with 100 µm crevices compared to 1 µm (p < 0.001) or 10 µm (p < 0.001) (Fig. 4c). In this case, no differences were noted for 1 µm (p = 0.4), 10 µm (p = 0.4), or 100 µm (p = 0.2) when changing the number of crevices. In Fig. 4, representative frames of the image produced by Verasonics, which includes color-Doppler overlayed on B-mode, and the image created using the magnitude of the saved IQ data for the cases of 100, 1 and 100 µm crevices are shown for each frequency. Representative frames of the control wafer at each frequency are also presented. As the wavelength of ultrasound at the investigated frequencies is much longer than the crevice sizes, crevices were not visible on B-mode.

### 4.3 Modeling crevice bubbles on silicon

**Fig. 5:**
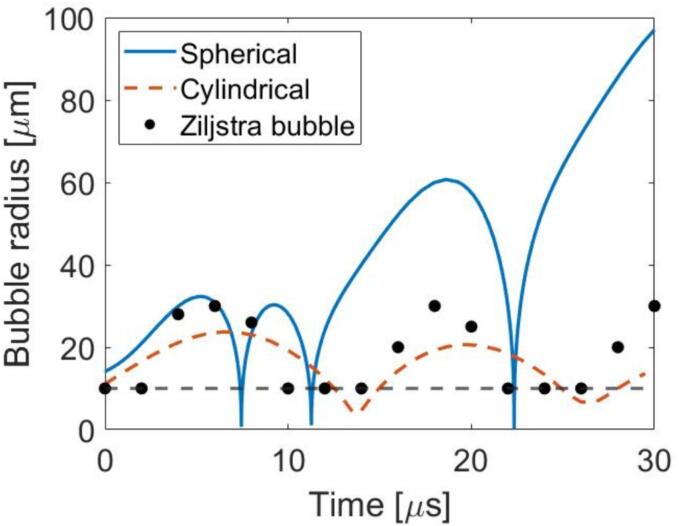
(color online) Comparison of spherical bubble model (blue solid), cylindrical bubble model (orange dotted), and the experimental results from Ziljstra et el. [36] (black circles). Any value at or below the crevice height (black dashed line) could not be visualized by the camera. All bubbles had radii of 30 µm and were driven at 0.08 MHz with an amplitude of 0.15 MPa. The cylindrical bubble model used a depth of 10 µm. (For interpretation of the references to color in this figure legend, the reader is referred to the web version of this article.)

When comparing the spherical (RP) and cylindrical (crevice) bubble models with the crevice bubble imaged in Ziljstra et al. (2015) [36] (Table 1 – column 2), the driven bubble matched better with the cylindrical bubble model (mean amplitude difference of 23 %) than the spherical model (mean amplitude difference of 172 %) (Fig. 5). The imaged bubble grew with a period of ∼12 µs which was similar to the cylindrical model where the bubble was predicted to grow with a period of ∼10 µs. The driving amplitude in the paper was not specified so an arbitrary value of 0.15 MPa was used. Different driving amplitudes caused slight variations in the maximum bubble radius and period of growth, but in all observed cases the cylindrical bubble model matched better than the spherical model. Further comparisons between the spherical and crevice model for a 10 µm crevice bubble driven at 0.75, 2.5, and 5.0 MHz are presented in supplemental Fig. 1.

**Fig. 6:**
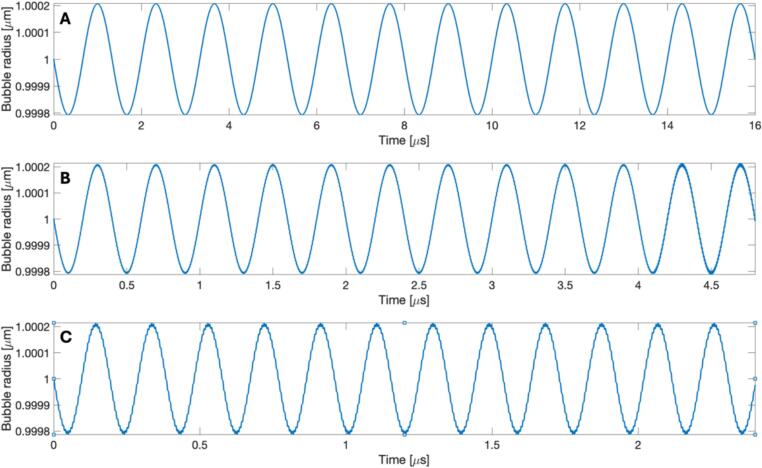
(color online) Results of cylindrical crevice bubble model showing simulated radial oscillations for the 1.2 μm bubble driven at (A) 750 kHz, (B) 2.5 MHz, and (C) 5.0 MHz withdriving amplitude (1 MPa), surface tension (3000 mN/m), crevice depth (1 µm), and pulse length (12 cycles) held constant.

When comparing the cylindrical crevice bubble model with the crevice bubbles on silicon imaged with high-speed photography (Table 1 – column 3), the experimental results matched well with the model when the surface tension used for the calculation was adjusted to the measured surface tension for the silicon wafer-water–air interface. For all 5 tested crevice sizes (0.8–1.2 μm) driven at three different frequencies (750 kHz, 2.5 MHz, 5.0 MHz), bubbles were only predicted to grow to a maximum radius of ∼0.05 nm. This would appear as no motion due to the resolution of the high-speed imaging. Representative examples are presented in Fig. 6 for the 1.2 μm bubble driven at 750 kHz, 2.5 MHz, and 5.0 MHz.

**Fig. 7:**
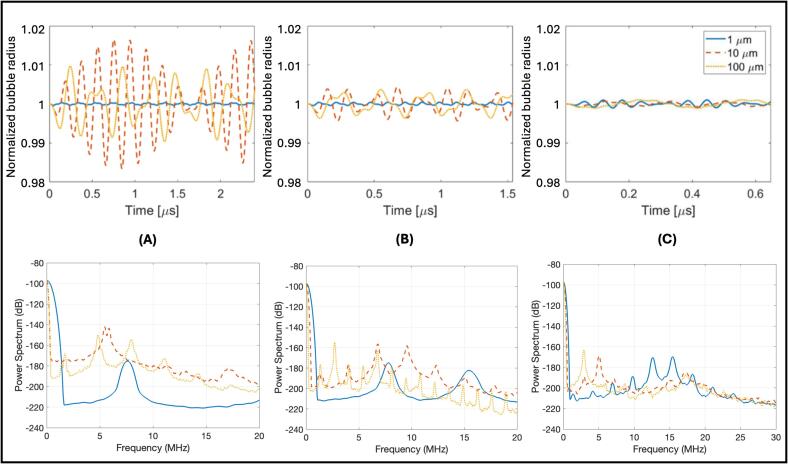
(color online) Results of cylindrical crevice bubble simulation for Doppler imaging parameters comparing crevice bubble radius over time and power spectra for each tested crevice size at (A) 5.0 MHz, (B) 7.8 MHz, and (C) 18.5 MHz. Driving amplitude (4 MPa), surface tension (3000 mN/m), crevice depth (10 µm) and pulse length (12 cycles) were held constant. The growth of the bubble radius on the y-axis is normalized to the crevice depth and refers to how the bubble expands into or out of the crevice.

When comparing the crevice bubble model to silicon wafers imaged with Doppler ultrasound (Table 1 – column 4), the larger amplitudes and crevice sizes compared to the previous wafers led to noticeable changes in bubble growth (Fig. 7). When driven at 5.0 MHz, the 10 µm diameter bubble was predicted to grow the most (∼0.2 µm) followed by the 100 µm diameter bubble (∼0.1 µm) and the 1 µm diameter bubble (∼0.002 µm). The 1 µm bubble only had one resonance peak which was shifted away from the driving frequency. The 10 µm bubble also only had one resonance peak located near the driving frequency. The 100 µm bubble oscillated at the driving frequency as well as other harmonic (2f, 3f, etc.), subharmonic (f/2, f/3, etc.), and ultraharmonic (3f/2, 5f/2, etc.) frequencies. Compared to 5.0 MHz, driving at 7.8 MHz caused the bubble growth to decrease to ∼0.04 µm for 10 and 100 µm diameter bubbles but increase to ∼0.004 µm for the 1 µm diameter bubble. The 1 µm bubble oscillated near the driving frequency and at harmonic frequencies while the 10 and 100 µm bubbles also oscillated at additional subharmonic and ultraharmonic frequencies. Finally, driving at 18.5 MHz caused the bubbles to only grow to motion to ∼0.01 µm in all cases. All three bubble sizes had less power in the driving frequency with the power distributed throughout subharmonic frequencies. Interestingly, the 1 µm bubble had larger power amplitudes at higher frequencies than the other tested crevice sizes. These wafers were not imaged in the high speed microscopy experiment. Further exploration of the crevice bubble model is presented in supplemental Fig. 2 for crevices ranging from 1-10000 µm.

## 5 Discussion

These results provide insight into how crevice bubbles may oscillate in an ultrasound field and the minimum size and number of crevices needed to cause twinkling on silicon wafers. Exposing 0.8–1.2 µm diameter crevice bubbles on silicon wafers to ultrasound at 750 kHz, 2.5 MHz, and 5.0 MHz and pressures up to 1 MPa did not elicit a visible response from the bubble on high-speed photography, which was validated by the computational model. Results from imaging 1–100 µm diameter cylindrical crevices etched into silicon showed that both the size and number of crevices affected twinkling. Notably, even 10 crevices as small as 1 µm produced measurable twinkling suggesting that perhaps even bubble oscillations of 0.002 µm is sufficient to cause twinkling.

Unlike previous studies visualizing bubbles in crevices, we observed no change in bubble size using high-speed photography. Previous studies observing bubbles on mineralizations either used a long negative lithotripter pulse (*p_+_* ≈ 3.2 MPa and *p_-_* ≈ 1.3 MPa) [15] or much lower frequencies (i.e. 416.5 kHz, p ≈ 1 MPa) [30] to enlarge the bubble for visibility at frame rates of 10–150 kfps and found bubbles to grow up to ∼50 µm. Furthermore, the low ultrasound pressures achieved in the small tank and high surface tension on the silicon wafer likely contributed to the bubble not growing. Indeed, the computational model shows that when the ultrasound pressures were increased or surface tension was reduced, large crevice bubble excursions were observed; however, we were unable to validate this experimentally. Further optical imaging driving the bubbles at higher pressures with a wider range of frequencies would provide further insight into their behaviour. While silicon was chosen because of the existing capabilities to etch micron sized crevices, it is important to note that there are important differences compared to more biologically relevant materials. For instance, mineralizations appearing in the body such as cholesterol, calcium phosphate, and uric acid have lower surface tensions than silicon [37], [38], [39] which may allow larger crevice bubble oscillations.

In general, as frequency increased, twinkling decreased, which agrees with previous work investigating the effect of frequency on twinkling [16], [40] and our computational model. Although the P4-2 transducer was not used for Doppler imaging in this study, it seems likely that the decrease in frequency would cause a similar increase in twinkling. While care was taken to keep conditions similar between the different tested frequencies, there will be inherent differences due to lower output pressures and higher attenuations at higher frequencies which will impact the results. Additionally, we did not explore the bubble response for driving frequencies other than the center frequency of each given transducer. At 5 and 7.8 MHz, 1 and 10 µm crevices produced more twinkling than 100 µm crevices. This result agrees with Rokni et al (2021) [16] where crevices with diameters of 1 µm on kidney stones were shown on environmental scanning electron microscopy (ESEM) to preferentially form bubbles. In contrast, at 18.5 MHz, 100 µm crevices twinkled the most compared to 1 and 10 µm crevices. Interestingly, the computational model predicted that the 10 and 100 µm crevice bubbles would grow larger than the 1 µm crevice bubbles when driven at 5 and 7.8 MHz while there was little difference in the amplitude of the bubble growth between crevice size when driven at 18.5 MHz. This result could suggest that larger bubble growth does not necessarily correspond to more twinkling.

When looking at the power spectra, the fundamental frequency of oscillation was not always exactly at the driving frequency. This frequency shift away from the driving frequency, was most notably present for the 1 µm crevices driven at 5 MHz. While it is not immediately obvious why this frequency shift occurs, understanding this phenomenon could provide insight into the relationship between the driving frequency and resonances of the cylindrical crevice bubbles. For example, increased damping has been shown to cause a frequency shift in the backscattered signal of microbubble contrast agents [41]. This additional damping could cause delays in the collapse of the bubble compared to the unbounded case, as observed in Fig. 5, which would lead to a shifted frequency response. Another possibility is that the added crevice boundaries could change the resonance compared to the unbounded case due to the increased stiffness [42]. The linear resonances for a spherical bubble can be calculated using the equation $f_{0}=\frac{1}{2\pi R_{0}}\sqrt{\frac{3\gamma P_{0}}{\rho_{0}}+\frac{2(3\gamma -1)\sigma }{\rho_{0}R_{0}}}$, where γ is the adiabatic index [43], resulting in resonances of 3 MHz, 0.3 MHz, and 0.03 MHz for 1, 10 and 100 µm radius bubbles, respectively. Assuming the boundaries would cause an upward resonance shift, then the driving frequencies would be much higher than the resonances of the 10 and 100 µm bubbles and lower than or near the resonance of the 1 µm bubble. Driving bubbles above versus below or equal to the resonance frequency would produce different frequency responses and could be another reason for the larger frequency shift for the 1 µm bubble compared to 10 or 100 µm. Future comparisons between the spectral content of the raw backscattered data and the model would provide further insight into the efficacy of this model and could give insight into the possible bubble shapes and oscillations for different crevice sizes or geometries.

It is important to note that different acoustic parameters were used for the photography and twinkling experiments. These differences arose because no bubble oscillations were observed in photography at the diagnostic ultrasound levels used in the twinkling experiment. This observation differed from our expectation, so we evaluated acoustic parameters that should maximize bubble growth. As bubble oscillation still remained elusive, we re-evaluated our computational model and found that when surface tension was modified to match the expectation for a silicon-water interface, no bubble oscillation should be expected. As twinkling was still evident, these data suggest that only the presence of bubbles, and not necessarily oscillation, was necessary for twinkling. While the model output represents bubble oscillations, twinkling is likely more closely associated with the backscatter from the oscillating bubble. Further simulations calculating this backscatter could provide more direct comparison between the model and experiments.

Although the crevice model matched the findings of Ziljstra et al. (2015) [36] better than the spherical model, there were differences in the period and amplitude that suggests the model does not perfectly encapsulate the conditions of the bubble. Ziljstra et al.’s (2015) [36] system was driven through base excitations and the actual driving amplitude could not be measured, which would impact the shape of the radius curve and contribute to the noted differences. One limitation to our derived model is that the bottom of the crevice is unaccounted for and could provide additional stiffness in the bubble. Additionally, this model only accounts for single bubble oscillation occurring in an infinite medium. Interactions between bubbles may have arisen in the multiple-crevice etchings which could have affected the resonance frequency [44], [45], [46] and bubble oscillation amplitudes [45], [46], [47], [48]. It is also possible that crevice bubbles are not cylindrically symmetric and could rather be conical, pyramidal, or form as a spherical bubble inside the crevice. Future experiments and modifications to the model are necessary to address how different bubble shapes affect the potential for oscillations and twinkling.

## 6 Conclusion

These results provide further insight into how crevice microbubbles cause twinkling. A modified crevice bubble model was derived and matched well with previous experimental results. Crevice bubbles on silicon wafers were driven with ultrasound and imaged with high-speed photography to visualize their dynamics, but no changes in the bubble radius were noted, which was verified by the computational bubble model. Finally, imaging silicon wafers with different numbers and sizes of cylindrical crevices with Doppler ultrasound produced twinkling in all cases, suggesting that even 10, 1 µm diameter crevices are enough to produce twinkling. Overall, these results provide a fundamental understanding of twinkling and could be used to better understand the intricacies of twinkling between different pathological mineralizations.

## CRediT authorship contribution statement

**Eric Rokni:** Writing – review & editing, Writing – original draft, Methodology, Investigation, Funding acquisition, Formal analysis, Conceptualization. **Eusila C. Kitur:** Writing – review & editing, Investigation. **Julianna C. Simon:** Writing – review & editing, Supervision, Funding acquisition, Conceptualization.

## Declaration of competing interest

The authors declare the following financial interests/personal relationships which may be considered as potential competing interests: Eric Rokni reports financial support was provided by National Science Foundation. Julianna C. Simon reports financial support was provided by National Science Foundation. If there are other authors, they declare that they have no known competing financial interests or personal relationships that could have appeared to influence the work reported in this paper.
