# Supplementary material for: Dynamics of crevice microbubbles that cause the twinkling artifact
Source: Ultrason Sonochem. 2024 Jun 25;108:106971. doi: 10.1016/j.ultsonch.2024.106971 (PMC11260575; doi:10.1016/j.ultsonch.2024.106971)
Supplement: Supplementary Data 1 [file mmc1.docx]

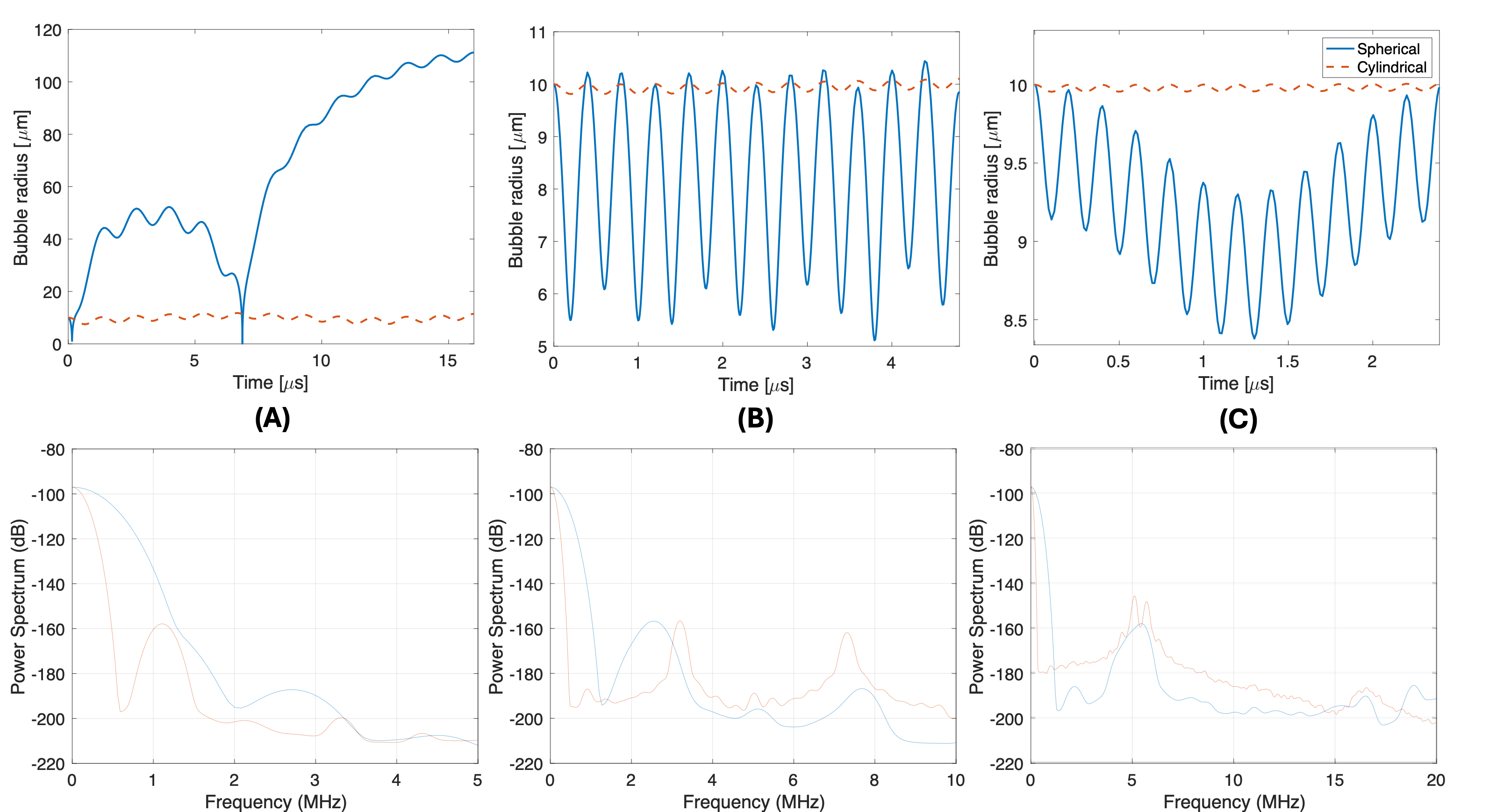


Supplemental Figure 1. (color online) Results of spherical and cylindrical crevice bubble model showing simulated radial oscillations and power spectra for a 10 µm diameters bubble driven at (A) 0.75 MHz, (B) 2.5 MHz, and (C) 5 MHz with driving amplitude (4 MPa), surface tension (3000 mN/m), crevice depth (10 µm), and pulse length (12 cycles) held constant.


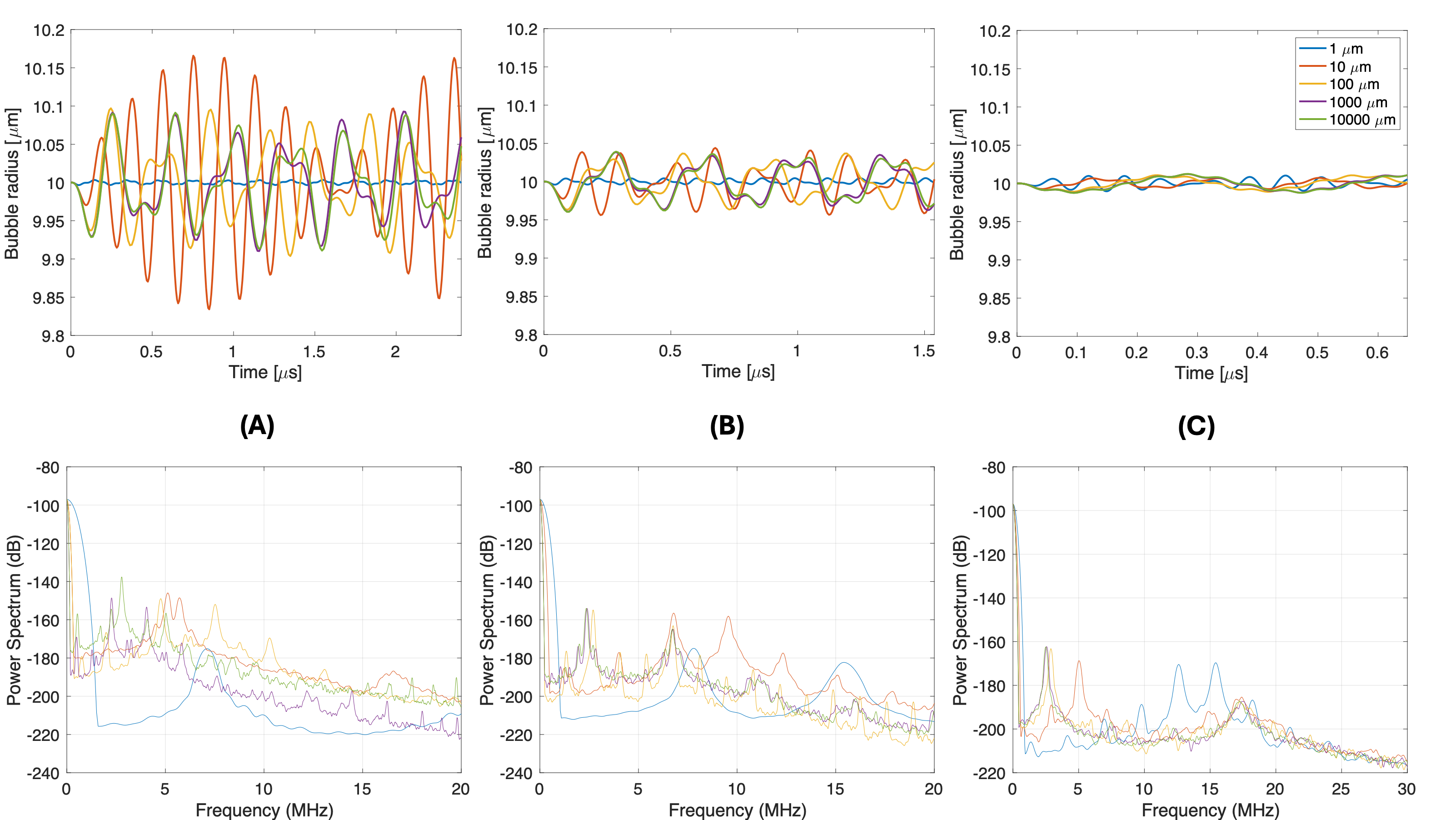


Supplemental Figure 2. (color online) Results of cylindrical crevice bubble model showing simulated radial oscillations and power spectra for a variety of bubble diameters driven at (A) 5 MHz, (B) 7.8 MHz, and (C) 18.5 MHz with driving amplitude (4 MPa), surface tension (3000 mN/m), crevice depth (10 µm), and pulse length (12 cycles) held constant. The growth of the bubble radius on the y-axis refers to how the bubble expands into or out of the crevice.
